# Beta-Hydroxybutyrate, Friend or Foe for Stressed Hearts

**DOI:** 10.3389/fragi.2021.681513

**Published:** 2021-06-08

**Authors:** Yuxin Chu, Cheng Zhang, Min Xie

**Affiliations:** ^1^ Department of Medicine, Division of Cardiovascular Disease, University of Alabama at Birmingham, Birmingham, AL, United States; ^2^ The Key Laboratory of Cardiovascular Remodeling and Function Research, Chinese Ministry of Education, Chinese National Health Commission and Chinese Academy of Medical Sciences, the State and Shandong Province Joint Key Laboratory of Translational Cardiovascular Medicine, Qilu Hospital of Shandong University, Jinan, China

**Keywords:** β-hydroxybutyrate, HDAC inhibition, ROS, myocardial ischemia/reperfusion injury, heart failure, cardiac metabolism, ketone bodies

## Abstract

One of the characteristics of the failing human heart is a significant alteration in its energy metabolism. Recently, a ketone body, β-hydroxybutyrate (β-OHB) has been implicated in the failing heart’s energy metabolism as an alternative “fuel source.” Utilization of β-OHB in the failing heart increases, and this serves as a “fuel switch” that has been demonstrated to become an adaptive response to stress during the heart failure progression in both diabetic and non-diabetic patients. In addition to serving as an alternative “fuel,” β-OHB represents a signaling molecule that acts as an endogenous histone deacetylase (HDAC) inhibitor. It can increase histone acetylation or lysine acetylation of other signaling molecules. β-OHB has been shown to decrease the production of reactive oxygen species and activate autophagy. Moreover, β-OHB works as an NLR family pyrin domain-containing protein 3 (Nlrp3) inflammasome inhibitor and reduces Nlrp3-mediated inflammatory responses. It has also been reported that β-OHB plays a role in transcriptional or post-translational regulations of various genes’ expression. Increasing β-OHB levels prior to ischemia/reperfusion injury results in a reduced infarct size in rodents, likely due to the signaling function of β-OHB in addition to its role in providing energy. Sodium-glucose co-transporter-2 (SGLT2) inhibitors have been shown to exert strong beneficial effects on the cardiovascular system. They are also capable of increasing the production of β-OHB, which may partially explain their clinical efficacy. Despite all of the beneficial effects of β-OHB, some studies have shown detrimental effects of long-term exposure to β-OHB. Furthermore, not all means of increasing β-OHB levels in the heart are equally effective in treating heart failure. The best timing and therapeutic strategies for the delivery of β-OHB to treat heart disease are unknown and yet to be determined. In this review, we focus on the crucial role of ketone bodies, particularly β-OHB, as both an energy source and a signaling molecule in the stressed heart and the overall therapeutic potential of this compound for cardiovascular diseases.

## Introduction

The human heart has an exceptionally high metabolic activity as a pump of the body, performing the daily work of circulating 7 tons of blood. It has been reported that the human heart can produce about 6 kg (almost 12 times of its own weight) of ATP daily (Bedi et al., 2016). The high ATP demand for the mechanical and electrical activities of the human heart is almost entirely fulfilled by oxidative phosphorylation, contributing up to 95% of the required ATP production, with the remaining 5% fulfilled by substrate-level phosphorylation in glycolysis (Gibb and Hill, 2018). In the normal adult heart, fatty acids serve as the primary fuel for mitochondrial ATP production, providing 40–70% of the ATP supply, followed by glucose (20–30%), lactate (5–20%), ketones (5–15%), and acetate, pyruvate and branched-chain amino acids (BCAAs) altogether contributing less than 5% to the needed ATP production (Gibb and Hill, 2018).

**FIGURE 1:**
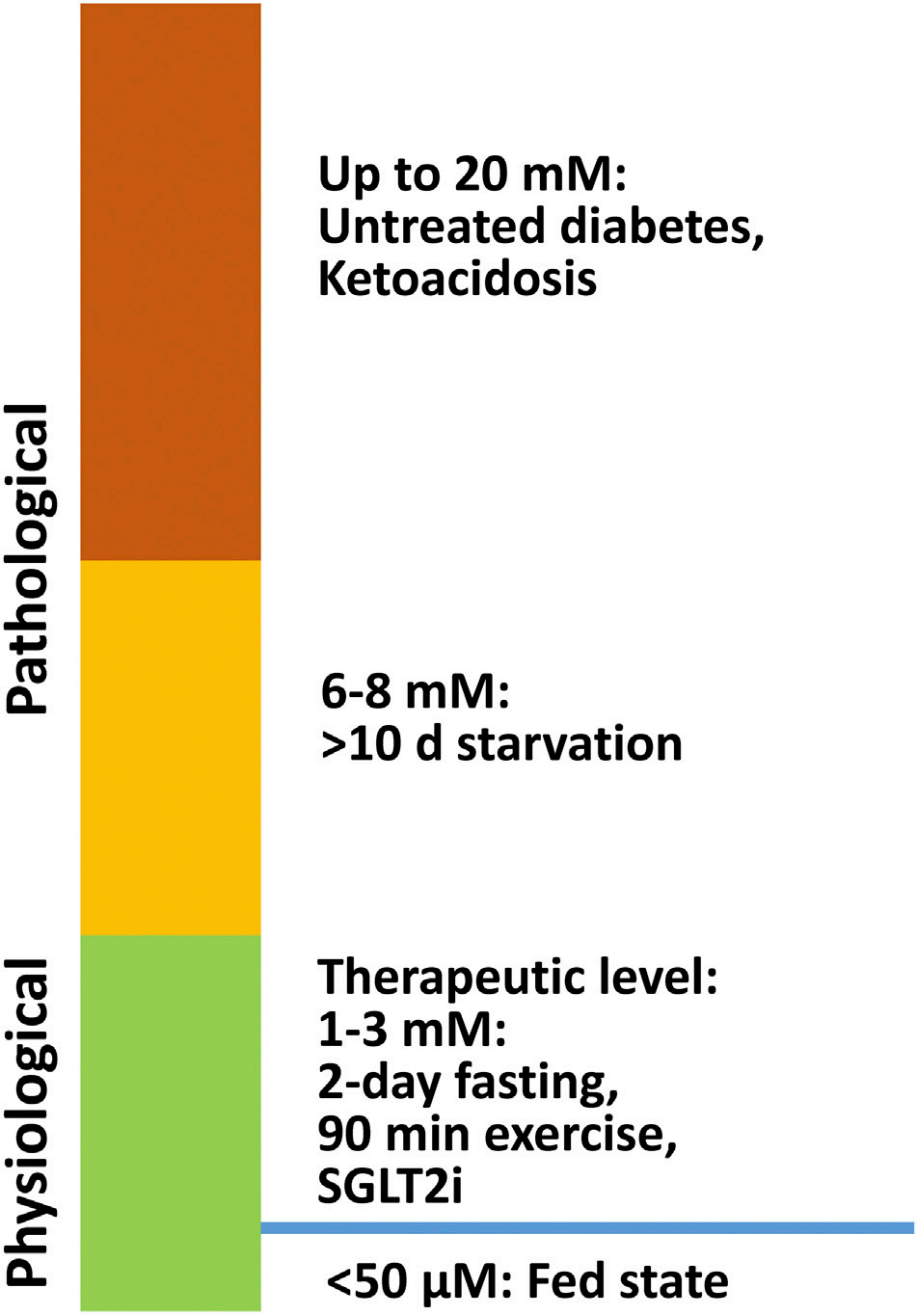
β-OHB levels under physiological and pathological conditions. In a normal fed state, the circulating ketone body concentration is approximately 50 μM, and it can increase to 1–8 mM in neonates, during fasting, after prolonged exercise, and on a ketogenic diet. In pathological conditions like diabetic ketoacidosis, ketone body concentration can accumulate up to 20 mM.

Ketone bodies, including acetoacetate, β-hydroxybutyrate (β-OHB), and acetone are small molecules synthesized mainly in the hepatic mitochondria and are transported to extrahepatic tissues like the heart, brain, and muscles for oxidation when fatty acid and carbohydrate availability is limited. Acetoacetate and β-OHB are the primary ketone bodies utilized by the heart (Cotter et al., 2013). In a normal fed state, the circulating ketone body concentration is approximately 50 μM. While in neonates, upon fasting, prolonged exercise, or following a ketogenic diet, it can raise up to 1–8 mM (Cahill, 2006; Cotter et al., 2013). In pathological conditions, such as diabetic ketoacidosis, the ketone body concentration can elevate to as high as 20 mM (Cotter et al., 2013) (Figure 1). In diabetes, as well as other disease conditions (e.g. heart failure), ketone body metabolism can also undergo some changes. However, the mechanism of increased ketogenesis during heart failure remains unclear (Aubert et al., 2016; Karwi et al., 2020).

Although most studies focus exclusively on β-OHB, the ketone body metabolism in the heart includes utilization of both β-OHB and acetoacetate, with the ratio of β-OHB to acetoacetate representing the mitochondrial NADH to NAD ratio (Chen et al., 2019). Acetoacetate’s utilization is also increased in diabetic rat hearts (Abdurrachim et al., 2019b), which can enhance contractile function via an anti-oxidation mechanism (Squires et al., 2003). Based on this observation, acetoacetate may serve as a prognostic marker for heart failure. It has been reported that high levels of acetoacetate correlate with increased mortality in patients with heart failure (Yokokawa et al., 2019), which is controversial to its previously reported protective roles. This may be explained by differences in disease states, including factors such as the severity of heart failure and the presence of diabetes (Yokokawa et al., 2019). Moreover, as compared to β-OHB, changes in acetoacetate levels are less affected by stress (Laffel, 1999), suggesting that β-OHB is a more stress-sensitive ketone body. Since the exact mechanism of the cardiac effect of acetoacetate is less well defined, in the present review we will focus only on the cardiac effects of β-OHB.

Under pathophysiological conditions, such as acute perturbations in workload or substrate availability, the heart exhibits a metabolic flexibility, changing its fuel utilization preference from one substrate to another. Aubert et al. used a quantitative proteomics approach to screen metabolic abnormalities in mouse models with impaired fatty acid utilization. The authors discovered that the levels of the proteins involved in fatty acid oxidation were reduced. In contrast, β-OHB dehydrogenase 1 (BDH1), an essential enzyme in the ketone body oxidation pathway, was upregulated in the compensated pressure overload-induced hypertrophic hearts as well as in decompensated failing hearts (Aubert et al., 2016). The myocardium is thought to be one of the highest ketone body consumers (Cotter et al., 2013), especially when the availability of other substrates is limited. Several studies have shown that there is an increased myocardial delivery and oxidation of ketone bodies as an alternative fuel source in human patients with advanced heart failure, increasing their proportion from less than 10% to over 20% (Wende et al., 2017; Ho et al., 2019; Horton et al., 2019). This shift is thought to be an early adaptive response to maintain adequate ATP production with reduced fatty acid oxidation.

However, whether the shift is adaptive or maladaptive remains controversial. The effects of increased levels of circulating ketone bodies are also not fully understood. This review provides an overview of the role of ketone bodies in the human heart upon stress and aims to unveil the potential of altering ketone body levels as a novel therapeutic approach for treatment of cardiovascular diseases.

## β-OHB Is Cardioprotective in Animal Models

Numerous studies have established that metabolic derangements can cause heart failure (Taegtmeyer et al., 2016; Wende et al., 2017). The myocardium of the failing heart exhibits a fetal-like metabolic profile, characterized by depressed fatty acid oxidation and increased glucose utilization (Taegtmeyer et al., 2016). Alterations in ketone body, amino acid, and protein metabolism have also been reported in the failing hearts (Wende et al., 2017). Many animal studies have focused on the cardioprotective effects of β-OHB as a stress reactive metabolite (Karwi et al., 2020).

**FIGURE 2:**
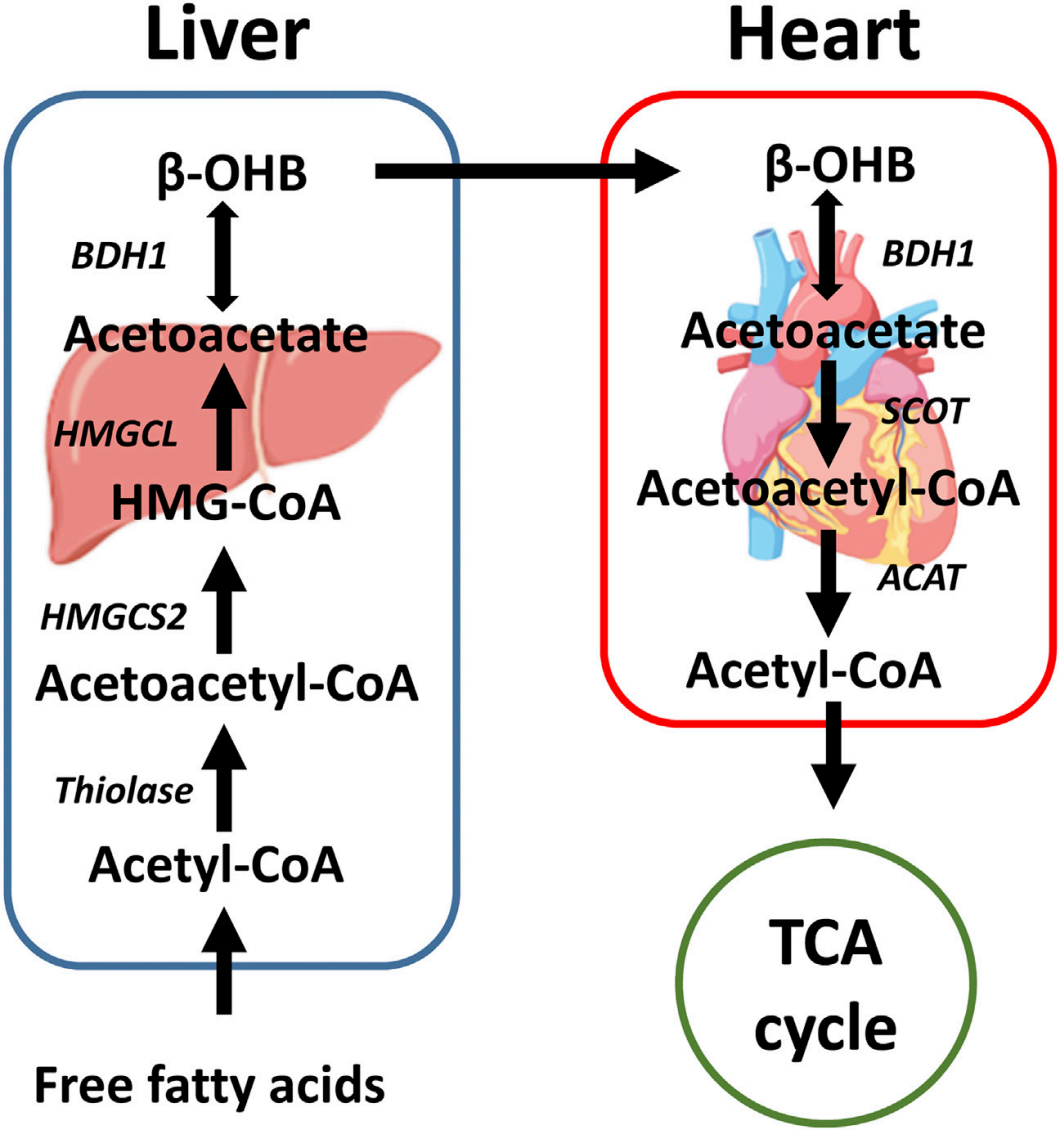
A brief schematic of the ketone body production and oxidation in the liver and heart. Ketone bodies are mainly produced in the liver with fatty acid oxidation. After being transported through the circulatory system, β-OHB can be oxidized in extrahepatic organs, such as the heart. In the heart, β-OHB can generate acetyl-CoA that can enter the TCA cycle. SCOT is the rate-limiting enzyme of ketone body oxidation. β-OHB: β-hydroxybutyrate, HMGCS2: 3-hydroxy-3-methylglutaryl-CoA synthase 2, HMG-CoA: 3-hydroxy-3-methylglutary-CoA, HMGCL: 3-hydroxy-3-methylglutaryl-CoA lyase, BDH1: β-hydroxybutyrate dehydrogenase 1, SCOT: Succinyl-CoA:3-oxoacid-CoA transferase, ACAT: Acetyl-CoA acetyltransferase, TCA cycle: Tricarboxylic acid cycle.

Under normal conditions, the utilization of β-OHB in the heart is low. However, ketone body utilization becomes essential to maintain metabolic homeostasis of the heart in pathological conditions. Increased utilization of ketone bodies can improve the prognosis of heart failure patients with diabetes (Rosenstein and Hough, 2016), while failure to use ketones can worsen prognosis (Rosenstein and Hough, 2016). Succinyl-CoA:3-oxoacid-CoA transferase (SCOT) and BDH1 are two enzymes that are required for terminal oxidation of β-OHB. BDH1 is responsible for the conversion of β-OHB to acetoacetate. SCOT is responsible for converting acetoacetate to acetoacetate-CoA, which is the rate-limiting step in ketone body oxidation. Notably, the conversions are dependent on the NAD+/NADH ratio and succinyl-CoA and succinate levels, which are reversible in the metabolic process (Cuenoud et al., 2020) (Figure 2). In a cardiomyocyte-specific SCOT knock-out mouse model, no apparent metabolic abnormalities were observed at the baseline, although ketosis was normally induced by fasting or ketogenic diets. However, after inducing pressure overload by transverse aortic constriction (TAC), the inability of the heart to oxidize ketone bodies due to the loss of SCOT resulted in significantly increased left ventricular volume and decreased left ventricular ejection fraction. This was accompanied by an increase in the myocardial reactive oxygen species (ROS), mitochondrial damage, and disruption of myofilament ultra-structure (Schugar et al., 2014). The importance of ketone body utilization has also been demonstrated in other mouse models. After being subjected to TAC and myocardial infarction (MI) surgery, the expression of BDH1 in failing hearts was significantly upregulated, indicating increased reliance on ketone bodies as a fuel (Aubert et al., 2016). Using a cardiac-specific BDH1 overexpression mouse model, the BDH1 transgenic mice showed no differences in the baseline characteristics. However, they were resistant to pathological cardiac remodeling under the stress of TAC, with limited impairment of cardiac function and attenuated cardiac fibrosis and hypertrophy. BDH1 overexpression can also reverse ROS-induced DNA damage and protein carbonylation as well as ameliorate mitochondrial ROS generation and apoptosis in failing hearts (Uchihashi et al., 2017). These protective effects may be attributed to increased utilization of β-OHB in the heart.

Because ketone bodies can regulate mitochondrial metabolism and ROS production, increasing delivery of β-OHB to the heart undergoing stress, such as ischemia/reperfusion (I/R) and heart failure after pressure overload, may have beneficial effects. Three days of fasting can significantly increase β-OHB concentration and effectively protect rat hearts from acute I/R injury (Snorek et al., 2012). Fasting can limit myocardial infarct size and reduce the occurrence of premature ventricular complexes as well as reperfusion-induced ventricular arrhythmias (Snorek et al., 2012). Zou et al. showed that after long-term fasting, high concentrations of β-OHB can reduce myocardial infarct size and apoptosis induced by I/R injury in rat models (Zou et al., 2002). Furthermore, the delivery of exogenous β-OHB can protect the heart from I/R injury. Continuously delivery of β-OHB before reperfusion can reduce infarct size and preserve cardiac function in I/R mice (Yu et al., 2018). It can also promote autophagic flux in the myocardium, reduce mitochondrial ROS formation, increase ATP production, attenuate mitochondrial swelling, and restore mitochondrial membrane potential (Yu et al., 2018). Mice incapable of utilizing β-OHB display worsened cardiac remodeling, while ketogenic chow can improve left ventricle remodeling in TAC/MI mice (Yurista et al., 2021). In tachycardia-induced myopathy canine models, β-OHB infusion can significantly preserve systolic function and ameliorate pathologic cardiac remodeling (Horton et al., 2019). β-OHB is not only available in IV forms but also oral salt and ketone ester (KE) forms, making it possible to increase β-OHB level orally (Lopaschuk et al., 2020). In mice and rats with heart failure, KE-enriched diets improved left ventricular systolic function and cardiac remodeling in both preventive and therapeutic protocols (Yurista et al., 2021). Altogether, these results indicate that moderately elevated β-OHB is cardioprotective during I/R and heart failure, possibly by enhancing energetic homeostasis.

However, some controversies still exist. One study reported that prolonged exposure to β-OHB had a detrimental effect on cardiomyocytes by altering glucose uptake and increasing ROS production via inhibiting the activation of AMPK/p38 MAPK signaling pathway (Pelletier and Coderre, 2007). This was also confirmed by other studies demonstrating that a prolonged exposure to β-OHB induced by deep fasting or frequent exogenous supplementation can inhibit mitochondrial biogenesis and lead to atrial fibrosis by activating Sirtuin 7 (Sirt7) transcription through inhibiting histone deacetylases 2 (HDAC2) (Xu et al., 2021). We have summarized the discrepancies of these studies in Supplementary Table S1, which underscores the importance of the timing and concentration of β-OHB given in different heart disease models, i.e., I/R injury, pressure-overload, diabetic cardiomyopathy. Thus, further studies are needed to optimize delivery of ketone bodies as a potential therapeutic for various heart diseases.

## β-OHB Is Cardioprotective in Humans

Heart failure is usually accompanied by metabolic derangement. Recently, many investigations have focused on ketone body metabolism and utilization in heart failure patients (Funada et al., 2009). Patients with metabolic disorders like diabetes have a higher level of ketone bodies during diabetic ketoacidosis (Figure 1), which is thought to be deleterious. However, in both diabetic and non-diabetic patients with severe heart failure, the utilization of ketones is increased (Lommi et al., 1996) and associated with the severity and prognosis of the disease (Chung et al., 2012; Marcondes-Braga et al., 2012; Yokokawa et al., 2016). It has been shown that in patients with end-stage heart failure, β-OHB utilization is significantly increased (Horton et al., 2019). BDH1 and BDH2, which are involved in the ketone body conversion back into acetoacetate for the tricarboxylic acid cycle, are also significantly increased (Aubert et al., 2016). Furthermore, the mRNA level of the rate-limiting enzyme SCOT essential for ketone body utilization increases in failing hearts (Bedi et al., 2016). There are two heart failure types, heart failure with reduced ejection fraction (HFrEF) and heart failure with preserved ejection fraction (HFpEF). The delivery of the exogenous 3-hydroxybutyrate (3-OHB, AKA β-OHB) to patients with HFrEF has beneficial hemodynamic effects by increasing cardiac output (Nielsen et al., 2019). These beneficial effects are detectable in the physiological concentration range (0.7–1.6 mM) of circulating β-OHB levels. Intriguingly, the hemodynamic effects of β-OHB were observed in both HFrEF patients and age-matched volunteers. These results suggest that β-OHB may be used as a potential therapy for HFrEF patients (Nielsen et al., 2019).

The treatment of HFpEF is concentrated on comorbidities management (Clark et al., 2021). HFrEF and HFpEF have different metabolic patterns. HFrEF patients had a similar or lower β-OHB level than HFpEF patients (Zordoky et al., 2015; Hunter et al., 2016). Corbi et al. showed that exercise training could increase Sirt1 activity and β-OHB level with improved antioxidant capacity in HFpEF patients (Corbi et al., 2019). Whether raising β-OHB blood levels in HFpEF patients has the same hemodynamic effects as in HFrEF patients is still unknown. Further studies are needed to clarify this.

Not surprisingly, fasting-induced ketogenesis or β-OHB supplements also prevent aging-related diseases (for example, neurodegenerative diseases, cardiovascular diseases, and cancers) and diabetes-related cardiovascular diseases. This topic was reviewed recently by Han et al. (Han et al., 2020). The mechanisms include weight loss, reduced triglycerides, LDL cholesterol, and blood glucose, and increased HDL cholesterol levels (Han et al., 2020). A randomized controlled human trial of one hundred participants carried out by Wei et al. showed that cycles of five-day fasting-mimicking diets were safe and effective in reducing markers and risk factors for aging and age-related diseases, including cardiovascular diseases (Wei et al., 2017). It was hypothesized that the reduction in serum glucose and IGF-1 contribute to the benefits. Also, the β-OHB level was significantly increased, shedding light on the β-OHB’s potential role in prevention of cardiovascular diseases (Wei et al., 2017). Although fasting-induced β-OHB is cardioprotective, a ketogenic diet with high-fat content might be detrimental in the long term. At least in rodents, a long-term ketogenic diet decreased sensitivity to peripheral insulin, impaired glucose tolerance (Kinzig et al., 2010), and induced atrial fibrosis (Xu et al., 2021). Thus, the intermittent fasting-mimicking diet cycles might be a promising lifestyle intervention to induce protective ketosis of β-OHB.

Nonetheless, increased β-OHB levels do not always correlate with positive clinical outcomes in human. Arrhythmogenic cardiomyopathy (AC) is a serious disease that may cause sudden death while lacking clinical biomarkers. A recent study demonstrated that elevated plasma β-OHB level might serve as a potential predictor of major adverse cardiovascular events (MACEs) in probands and disease progression in patients with AC and their clinically asymptomatic relatives (Song et al., 2020). It has also been reported that, as compared with normal controls, the atrial samples from patients with atrial fibrillation exhibit increased levels of ketone bodies (Xie et al., 2016).

The ability to switch to ketone bodies as energy source in stressed hearts is independent of the patient’s diabetes status (Aubert et al., 2016; Bedi et al., 2016). It is, however, still uncertain whether the enhanced cardiac ketone body utilization is the cause or the result of cardiac dysfunction. Thus, whether exogenous induction of ketosis is beneficial in all types of cardiovascular diseases requires further exploration. It is also challenging to deliver ketone bodies safely and precisely to regulate abnormal metabolism. More studies are needed to demonstrate the relationship between β-OHB and heart disease in humans and the best timing and dose of ketone body preparations to be delivered to them.

## Mechanisms Underlying the Biological Effects of β-OHB in the Heart and Other Organs

### Oxidative Roles

Ketogenesis is vital in cardiac health, especially during stresses, such as heart failure. The ketone bodies are generated in the liver, and the heart can use ketone bodies at baseline as a minor energy source and increase ketone body usage upon heart failure (Figure 2). In the failing hearts, β-OHB serves as an alternative fuel, providing more ATP than glycolysis without increasing cardiac efficiency (Lopaschuk et al., 2020). These oxidative roles of β-OHB have been reviewed recently (Lopaschuk et al., 2020). In this review, we will focus more on the non-oxidative roles of β-OHB in the heart.

### Non-Oxidative Roles

Although there are many studies showing cardioprotective effects of β-OHB in addition to its oxidative roles, the underlying specific mechanisms of cardioprotection remain elusive. Elevated β-OHB has been found as an effective treatment for epilepsy (Martin et al., 2016) and played a neuro-protective role in neurodegenerative diseases, such as Parkinson’s disease (Tieu et al., 2003) and Alzheimer’s disease (Shippy et al., 2020). The possible mechanisms of β-OHB’s neuroprotective effects include mitochondrial metabolism regulation (Li et al., 2021), inhibition of the mammalian target of rapamycin (mTOR) pathway (Thau-Zuchman et al., 2021), and glutamatergic excitatory synaptic transmission reduction (Lund et al., 2009). In addition, β-OHB has also been reported to contribute to cancer cell stemness by regulating cell proliferation and facilitating key transcriptional factor expression (Wang et al., 2019; Ji et al., 2020). These functions suggest that β-OHB is not only serving as an alternative fuel but also as a signaling molecule.

#### β-OHB Is an Endogenous HDAC Inhibitor and Maintains Mitochondrial Homeostasis

Histone deacetylases (HDACs) play essential roles in regulating gene transcription by balancing the acetylation activities of histone acetyltransferases (HATs). Acetylation is an essential post-translational modification of many proteins, which regulates mitochondrial metabolism and function (Felisbino and Mckinsey, 2018). Acetylation of ε-lysine groups can lead to chromatin structure relaxation, thus enhancing the DNA binding proteins’ accessibility and increasing transcriptional activation (Wang et al., 2014). Since HDACs modify the lysine residue in other proteins to affect their function, HDACs are also referred to as lysine deacetylases (KDACs). According to their homology to yeast transcriptional repressors, HDACs can be divided into four classes. Class I includes HDACs 1, 2, 3, and 8; Class IIa includes HDACs 4, 5, 7, and 9; Class IIb includes HDACs 6 and 10; Class IV includes HDAC 11, which are Zn^2+^ dependent for enzymatic activity and Class III, also called Sirt1-7, uses a different NAD + dependent mechanism to regulate their activity (Gregoretti et al., 2004). The activity of HATs is dependent on nuclear acetyl-CoA concentrations (Felisbino and Mckinsey, 2018).

It has been reported that HDACs activity is elevated in models of heart failure, diabetic heart, and myocardial I/R injury (Granger et al., 2008; Nural-Guvener et al., 2014; Evans and Ferguson, 2018; Bocchi et al., 2019). Class I and II HDAC inhibitors represent a group of small molecule epigenetic modifiers that have demonstrated efficacy in animal models of heart failure over the last decade (Evans and Ferguson, 2018). HDAC inhibition has also been used as a therapeutic strategy for cardiac I/R injury (Xie et al., 2019). Emerging evidence also suggests that inhibition of HDACs protects the heart against myocardial injury and stimulates endogenous angiomyogenesis even in the diabetic heart (Bocchi et al., 2019). Thus, inhibition of HDACs using small molecules has been thought to be a promising approach to treat many pathological disorders.

Multiple pathways have been implicated in the cardioprotective effects of HDAC inhibitors, including apoptosis, inflammation, metabolism, and autophagy (Xie et al., 2014; Schotterl et al., 2015; Das Gupta et al., 2016). β-OHB is an endogenous Class I HDAC inhibitor that can cause an increase in histone acetylation (Shimazu et al., 2013). In human embryonic kidney 293 (HEK293) cells, β-OHB can significantly increase the acetylation levels of histone H3 lysine 9 and histone H3 lysine 14. *In vitro*, β-OHB inhibits the activity of HDAC 1, 3, and 4, indicating that it is a Class I HDAC inhibitor. This inhibitory effect is through increasing histone acetylation at the FOXO3a promoter in HEK293 cells (Shimazu et al., 2013). Physiologically achievable millimolar concentrations of β-OHB can significantly increase histone acetylation through HDAC inhibition directly (Shimazu et al., 2013). However, inhibition of HDAC 6, one of the Class II HDACs, shows no benefit and is even detrimental to cardiomyocytes during I/R injury (Aune et al., 2014). Another study shows that a pretreatment with suberoylanilide hydroxamic acid (SAHA), a second-generation HDAC inhibitor, effectively protects murine fibrosarcoma L929 cells against TNF-α induced necroptosis (Wang et al., 2013). This mechanism may include induction of multiple transcription factors that can initiate cell-protective autophagy.

Since HDAC inhibitors can induce autophagy in multiple cell types, including cardiomyocytes, it will be essential to test whether β-OHB’s cardioprotective effects are mediated through autophagy. Autophagy is a lysosomal degradation pathway that plays an essential role in removing protein aggregates and damaged organelles to maintain homeostasis (Kroemer et al., 2010). In nutrient deprivation status, autophagy is thought to be an adaptive response to recycle cytosolic elements, thus providing substrates for energy metabolism (Kim et al., 2007). Autophagy has also been shown to play an essential role in mitochondrial metabolism, especially in cardiomyocytes. During reperfusion, autophagy is blocked and contributes to the reperfusion injury following ischemia (Ma et al., 2012). Dian J. Cao and his colleagues showed that autophagy is an obligatory element in cardiac hypertrophic remodeling and HDAC 1 and 2 are required effectors (Cao et al., 2011). β-OHB is a known endogenous inhibitor of HDAC1 and 2 (Shimazu et al., 2013).

Mitophagy is a specialized autophagy mainly responsible for the clearance of damaged mitochondria by lysosomes (Whitworth and Pallanck, 2017). This is important in ischemic cardiovascular diseases because damaged mitochondria are a source of oxidative stress, which can affect mitochondrial function, thus leading to cellular degeneration in the disease status (Bingol et al., 2014). In aged rabbit hearts, β-OHB can enhance mitochondrial quality control by activating Parkin translocation while worsening mitophagy with impaired mitochondrial fission and fusion upon heart failure (Thai et al., 2019). β-OHB has also been shown to reduce oxidative stress during I/R (Shimazu et al., 2013). As a crucial part of normal and pathophysiological metabolism, β-OHB has profound effects on various mitochondrial functions, such as reducing the mitochondrial NAD couple and oxidizing coenzyme Q (Veech, 2014; Xie et al., 2016). Superoxide dismutase 2 (SOD2) is an enzyme located within the mitochondrial matrix and works as a superoxide scavenger. Under H_2_O_2_ stimulation, β-OHB can increase FOXO3a expression level, which can induce antioxidant enzymes SOD2 and catalase, suggesting that β-OHB can reduce ROS generation in oxidative status (Nagao et al., 2016). A short-term ketogenic diet increases mitochondrial abundance in the myocardium, partially explaining the protective effect of β-OHB (Al-Zaid et al., 2007).

In diabetic patients the prevalence and severity of cardiovascular diseases are significantly increased, compared with non-diabetic patients, accompanied by a much worse prognosis. Chen et al. showed that in diabetic hearts, HDAC inhibition plays a critical role in improving cardiac function and suppressing myocardial remodeling and is associated with decreased apoptosis, stimulation of endogenous angiogenesis, increase of anti-oxidant SOD1 and activations of GLUT1 acetylation and p38 phosphorylation (Chen et al., 2015). Since β-OHB concentrations are always kept at high levels in both type 1 and 2 diabetic patients, it is intriguing to explore the role of β-OHB in diabetic patients also suffering from a cardiovascular disease.

In summary, many cardiac stresses, such as cardiac I/R injury and diabetes, can induce HDAC activity, which contributes to the pathogenesis of heart failure. HDAC inhibition has been shown to be effective in preventing cardiac I/R injury and improve the function of a failing heart. These are probably through inducing autophagy/mitophagy. β-OHB is an endogenous HDAC inhibitor. However, whether the beneficial effects of β-OHB during cardiac I/R are exerted through regulation of the autophagy/mitophagy pathway as well as mitochondrial functions via HDAC inhibition, still needs more investigation.

#### β-OHB Is an Endogenous NLRP3 Inflammasome Inhibitor

The Nod-, LRR- and pyrin domain-containing protein 3 (Nlrp3) inflammasome is an important innate immune sensor acting through regulation of the caspase-1 activity and the release of proinflammatory cytokines (Lamkanfi and Dixit, 2014). Suppression of Nlrp3 inflammasome can ameliorate the progression of atherosclerosis (Duewell et al., 2010), diabetes (Ding et al., 2019), Alzheimer’s disease (Heneka et al., 2013), and other functional declines related to aging (Camell et al., 2019). Inflammation is an unmissable component of the heart stress. Numerous studies have highlighted the role of Nlrp3 inflammasome during the I/R injury event (Toldo and Abbate, 2018). β-OHB is an endogenous inhibitor of the Nlrp3 inflammasome, attenuating inflammatory responses (Yamanashi et al., 2017). In high salt-fed hypertensive rats there is a lower level of circulating β-OHB, while a nutritional supplementation of the β-OHB precursor, 1,3-butanediol, rescues kidney function, resulting in nearly normal blood pressure. The therapeutic effects of 1,3-butanediol are comparable to that of exercise. These beneficial effects are likely to be exerted through inhibition of the renal Nlrp3 inflammasome (Chakraborty et al., 2018). Another study found that the anti-inflammatory effect of ketogenic diet may be attributed to the β-OHB-mediated inhibition of Nlrp3 inflammasome formation (Youm et al., 2015). Increasing the circulating β-OHB levels by SCOT knockout in skeletal muscle reduced cardiac Nlrp3 inflammasome activation upon heart failure (Byrne et al., 2020). Endoplasmic reticulum (ER) stress can induce Nlrp3 inflammasome. Bae et al. showed that β-OHB can inhibit both ER stress and Nlrp3 inflammasome activity in rats through the AMPK activation (Bae et al., 2016). In HFpEF mice, β-OHB can delay the progression of HFpEF probably by attenuating Nlrp3 inflammasome formation and proinflammatory cytokines to prevent mitochondrial dysfunction (Deng et al., 2021).

Therefore, in addition to providing energy during heart failure and I/R, β-OHB may also reduce detrimental inflammatory responses. Thus, inhibiting Nlrp3 inflammasome formation can have positive effects, such as reduction of infarct size and prevention of heart failure. Since drugs like MCC950 are still in the development stage (van Hout et al., 2017), there is no selective Nlrp3 inflammasome inhibitors currently available for clinical use. Alternatively, as an endogenous Nlrp3 inflammasome inhibitor, increasing the circulating β-OHB levels may be used to treat Nlrp3 inflammasome-related cardiovascular diseases, such as atherosclerosis, coronary heart diseases, and heart I/R injury (Tong et al., 2020).

#### β-OHB Is an Endogenous Ligand of GPRs

β-OHB is an endogenous ligand for a nicotinic acid receptor G protein-coupled receptor (GPR) 109A (Offermanns et al., 2011), also known as hydroxycarboxylic acid receptor 2 (HCAR2). Its specific binding can activate the adipocyte-expressed GPCRs, HM74a/PUMA-G, to reduce lipolysis in adipocytes and regulate fatty acid availability and metabolism (Tunaru et al., 2003; Taggart et al., 2005), which may have anti-atherosclerosis and anti-inflammatory effects (Lukasova et al., 2011). This suggests that activation of HCAR2 may be a potential therapeutic target for the treatment of diseases, such as atherosclerosis. β-OHB can also affect cardiovascular function via another GPCR family member, GPR41. It has been reported that β-OHB administration can decrease sympathetic outflow and heart rate through antagonizing GPR41 (Kimura et al., 2011), thereby maintaining metabolic homeostasis via the GPR41-SNS pathway (Kimura et al., 2011). The role of GPRs in ischemic neurological diseases have been well established. However, whether β-OHB can protect hearts from I/R injury via GPRs still needs to be determined. Therefore, further studies are needed to unveil the relationship between β-OHB and GPRs in cardiovascular diseases.

#### Other Non-oxidative Roles of β-OHB

In addition to acting as an HDAC inhibitor, Nlrp3 inflammasome inhibitor, and GPR ligand, ketone bodies have other roles that are likely to impact the cardiovascular system. As low as 0.5 mM β-OHB can improve cardiac cell excitation-contraction coupling under hypoxia, providing evidence that β-OHB can exert cardioprotective benefits under stress (Klos et al., 2019). β-OHB can also reduce oxidative stress and induce anti-aging effects through binding to hnRNP A1 to upregulate Oct4 expression at the concentration of 4 mM in the vascular system (Han et al., 2018). Besides enhancing gene expression by inhibiting HDACs, β-OHB can also promote protein hyperacetylation via increasing cellular acetyl-CoA levels to maintain metabolic balance (Newman and Verdin, 2014). Lysine β-hydroxybutyrylation (Kbhb) is a post-translational modification of histone induced by increased β-OHB levels, which can activate gene transcription and cell proliferation (Xie et al., 2016). It has been reported that Kbhb is involved in the upregulation of genes in the starvation-responsive metabolic pathways (Xie et al., 2016) and β-OHB may become a new target for cancer treatment through regulating p53 activity (Liu et al., 2019). These observations suggest that β-OHB’s cardiac protective effects may be achieved through other epigenetic mechanisms other than HDAC inhibition. Poly-R-β-hydroxybutyrate (PHB) is a polymer of β-OHB and a part of the mitochondrial membrane system (Dedkova and Blatter, 2014). It can increase mitochondrial membrane permeability by regulating the opening of the permeability transition pores required for cardiovascular metabolism and apoptosis pathways (Dedkova, 2016). These studies demonstrate that β-OHB plays an essential role in multiple pathways in non-cardiomyocytes, warranting more studies to elucidate its function in cardiovascular diseases.

## SGLT2 Inhibitors can Increase the Production of β-OHB

The sodium-glucose cotransporter-2 inhibitors (SGLT2i) are diabetic medications to control blood glucose. SGLT2i targets renal glucose reabsorption in an insulin-independent manner, which can induce glycosuria of 60–90 g/day (Tahrani et al., 2013). Unlike the traditional antidiabetic agents, SGLT2i offers unique benefits to type 2 diabetic patients with high cardiovascular risks. Many clinical studies have been carried out to show remarkable cardiovascular outcomes in patients using SGLT2i (Zinman et al., 2015; Neal et al., 2017; Wiviott et al., 2019).

The SGLT family is composed of seven isoforms: SGLT1-6 and Sodium-myo-inositol co-transporter 1 (SMIT1). SGLT1 has the highest affinity for glucose in the gastrointestinal tract and is also involved in kidney and cardiac glucose handling (Wright et al., 2011). In human heart, GLUT4 is the major isoform that represents approximately 70% of the total glucose transporters. Expression of SGLT1 is increased in patients with end-stage cardiomyopathy secondary to type 2 diabetes. Increased expression of SGLT1 is a compensatory mechanism to the reduction in cardiac GLUT1 and GLUT4 expression (Szablewski, 2017). Although SGLT1 is thought to be an important cardiac glucose transporter in type 2 diabetic patients (Szablewski, 2017), a recent study demonstrated that phlorizin, a dual SGLT1/2 inhibitor, can reduce cardiomyocyte glucose uptake independent of SGLT1, suggesting that SGLT1/2 inhibitor might inhibit other glucose transporters (Ferte et al., 2021). In diabetic mice, inhibition of SGLT1 using shRNA, could alleviate inflammation and pyroptosis induced by hyperglycemia (Sun et al., 2021). To investigate whether using a dual SGLT1/2 inhibitor can enhance the protective effects of SGLT2i, Bode et al. carried out a study in mouse HFpEF models and found that the SGLT1/2 inhibitor sotagliflozin can improve atrial remodeling and exhibit anti-arrhythmic effects (Bode et al., 2021). A recent study showed that the dual SGLT1/2 inhibitors may protect patients with diabetes from MI and stroke better, while the beneficial effects in patients without diabetes require more evaluation (Pitt and Bhatt, 2021). SGLT2 is the main co-transporter in humans that helps reabsorbing 80–90% of the glucose filtered by the glomerulus (Bonora et al., 2020), and SGLT1 helps reabsorbing additional renal glucose that evades SGLT2 (Bertrand et al., 2020). However, since the SGLT2i are available as therapeutic agents, most of the current clinical results reviewed are from SGLT2i.

In a clinical trial involving 7,020 patients, compared with placebo, empagliflozin showed a lower rate of primary composite cardiovascular outcome and all-cause mortality in patients with type 2 diabetes (Zinman et al., 2015). Neal et al. showed similar results that canagliflozin can lower the risk of cardiovascular events with a greater risk of amputation at the level of the toe or metatarsal (Neal et al., 2017). Wiviott et al. showed that treatment with dapagliflozin resulted in a lower rate of cardiovascular death or hospitalization for heart failure in patients with type 2 diabetes (Wiviott et al., 2019). Recent data from DAPA-HF (Dapagliflozin and Prevention of Adverse Outcomes in Heart Failure), EMPEROR-Reduced (EMPagliflozin outcomE tRial in Patients With chrOnic heaRt Failure With Reduced Ejection Fraction) and EMPA-TROPISM (Are the “Cardiac Benefits” of Empagliflozin Independent of Its Hypoglycemic Activity) showed the benefit of SGLT2i in both diabetic and non-diabetic heart failure patients (Mcmurray et al., 2019; Packer et al., 2020; Santos-Gallego et al., 2021). Dapagliflozin can reduce the risk of cardiovascular death or heart failure exacerbation in HFrEF patients shown in the DAPA-HF trial (Mcmurray et al., 2019). Empagliflozin significantly improves cardiovascular and renal outcomes in patients independent of baseline diabetes status in the EMOEROR-Reduced trial (Packer et al., 2020) and significantly improves cardiac function and quality of life in HFrEF patients in the EMPA-TROPISM trial (Santos-Gallego et al., 2021). The clinical trial to investigate the effects of SGLT2i on HFpEF is still in progress (Anker et al., 2020).

Although the beneficial effects of SGLT2i in heart failure can be attributed to improved volume status, glucose-lowering, etc., the exact mechanisms of achieving the cardiac benefits of SGLT2i are not fully delineated (Santos-Gallego et al., 2021). SGLT2i can induce a persistently low level of ketone body production, mainly β-OHB. Mechanistically, SGLT2i decreases the level of glucose in the blood and increases the production of glucagon, which promotes ketogenesis. On the other hand, SGLT2 receptors present on the surface of pancreatic α-cells can act as glucose sensors (Bonner et al., 2015). Furthermore, SGLT2i can increase lipolysis and the subsequent production of ketone bodies in the liver (Taylor et al., 2015). Ketone bodies use less oxygen to generate the same amount of ATP as glucose and fatty acids (Puchalska and Crawford, 2017). Thus, the diabetic hearts can benefit from the SGLT2i treatment. Mildly elevated ketone levels can promote fuel efficiency and reduce ROS formation, which might improve cardiac function and the prognosis of heart failure (Oh et al., 2019). In addition to inducing ketosis, SGLT2i can mediate other effects, like the natriuretic effect, plasma volume contraction, to reduce cardiac preload. Also, through lowering blood pressure and arterial stiffness, cardiac afterload can be reduced (Selvaraj et al., 2020). In addition, SGLT2i can attenuate Nlrp3 inflammasome activation in isolated macrophages, which has also been proved in diabetic patients with cardiovascular diseases (Kim et al., 2020). Since SGLT2i′s targets are mainly located on the renal proximal tubular epithelium, SGLT2i mediated natriuresis may account for its hemodynamic improvement effect (Sano, 2017). To what degree SGLT2i′s cardiac protection is mediated by β-OHB remains challenging to clarify. Verma et al. showed that empagliflozin, or β-OHB, can enhance cardiac metabolism in diabetic mice by increasing ATP production rates without affecting cardiac efficiency (Verma et al., 2018). In a study using a novel ketone probe and magnetic resonance spectroscopy, empagliflozin was shown to be able to increase circulating β-OHB levels but not myocardial ketone body utilization in spontaneously hypertensive heart failure (Abdurrachim et al., 2019a). This suggests that SGLT2i may be beneficial to the heart, mainly via β-OHB through its non-oxidative functions. Furthermore, SGLT2i can improve glycemic regulation via an insulin-independent mechanism (Ferrannini and Solini, 2012). The therapeutic application of SGLT2i could lead to weight loss (Merovci et al., 2014), correction of hyperinsulinemia (Matsubayashi et al., 2020), and reduced hyperleptinemia (Vickers et al., 2014). SGLT2i could also modulate inflammation via IL-1β (Nakatsu et al., 2017) and Nlrp3 inflammasome (Sukhanov et al., 2021). These features together contribute to SGLT2i′s therapeutic effects in patients with heart failure.

Among multiple classes of antidiabetic agents, SGLT2i showed significant cardiovascular benefits, which might be attributed, at least partially, to its ability to increase the concentration of ketone bodies, especially β-OHB. SGLT2i is beyond its initial objective as an antidiabetic drug and the ongoing studies could further uncover the underlying molecular mechanisms. With a better understanding of the mechanism, we are on track to develop novel heart failure treatments in patients with or without diabetes.

## Potential Therapeutic Applications of β-OHB

Given the benefits of exogenous β-OHB in patients with heart failure, several therapeutics have been developed to deliver β-OHB. β-OHB infusion in patients with HFrEF can significantly improve cardiac output and reduce systemic vascular resistance in a dose-dependent manner without impairing the myocardial external energy efficiency (Nielsen et al., 2019). Oral forms of β-OHB include 1,3-butanediol (Scott et al., 2019), medium-chain triglyceride (MCT) supplementation (Brunner et al., 2021), KEs, and ketone salts (Stubbs et al., 2017). Among them, KEs are the most promising therapeutics, with smaller effective doses and fewer side effects. KEs are typically comprised of several ketones or ketogenic precursors, and their decomposition products can be directly absorbed into blood as ketone bodies or their precursors (Clarke et al., 2012). Several clinical trials have been carried out to test KEs’ effects in healthy and cardiovascular disease conditions. KEs can reduce blood glucose, free fatty acids, and triglyceride levels (Stubbs et al., 2017), as well as lower plasma insulin, ghrelin, glucagon-like peptide-1, and peptide tyrosine levels in healthy adults (Stubbs et al., 2018). More ongoing studies are testing the effect of KEs in pre-diabetic patients. These studies will determine whether regulating ketone levels can become a promising therapeutic strategy in diseases with metabolic disorders like diabetes and heart failure.

## Conclusion and Perspectives

**FIGURE 3:**
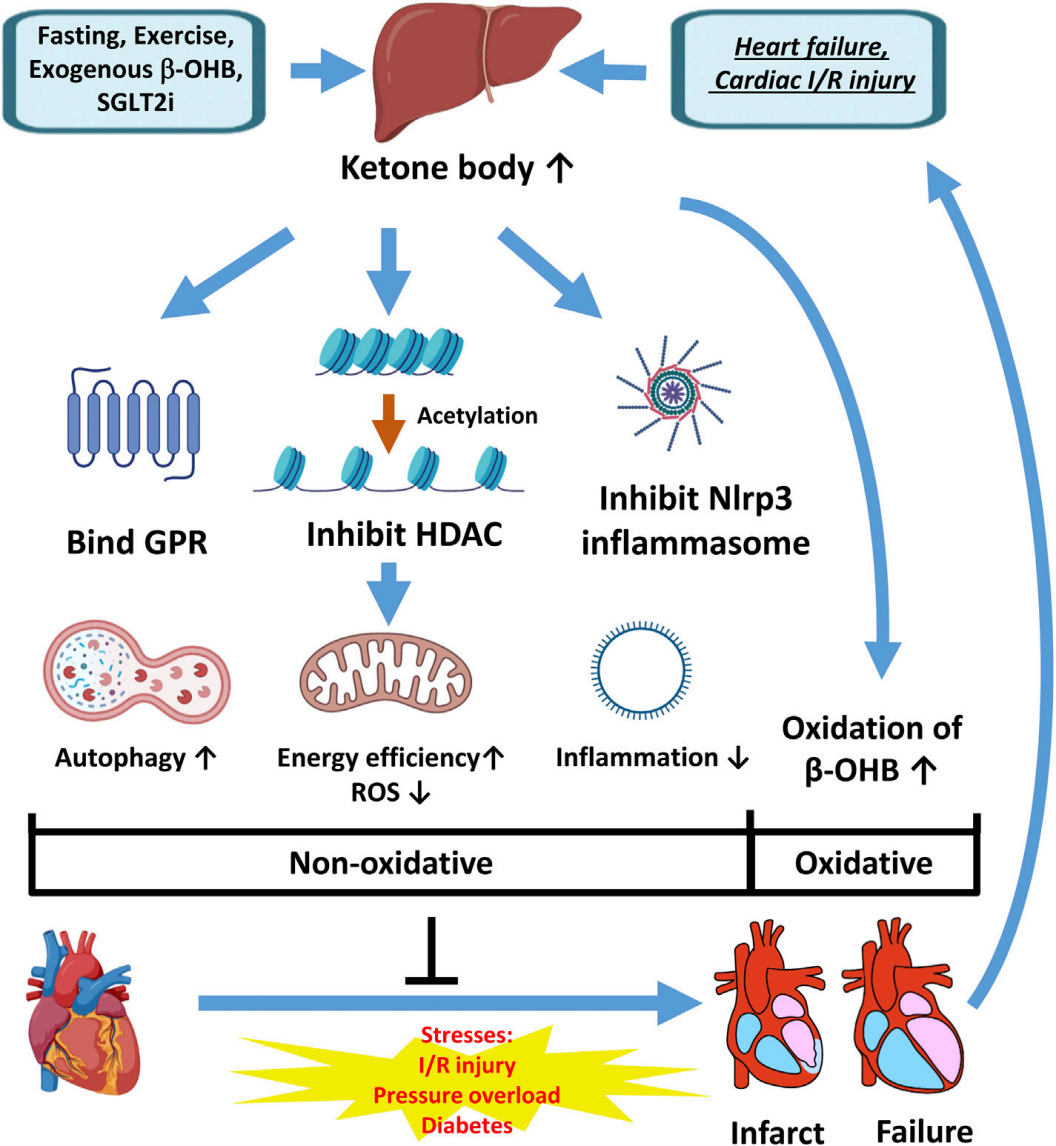
The beneficial effects of β-OHB during the cardiac stresses. The concentration of β-OHB is increased after prolonged fasting, long-time exercise, exogenous supplementation, SGLT2i therapy, and more importantly, as feedback from heart failure and I/R injury. As a result, the utilization of β-OHB is increased in the failing hearts and the hearts subjected to I/R injury as an alternative fuel to protect the myocardium (oxidative effect). In addition, as a ligand for GPR, an HDAC inhibitor, and an Nlrp3 inflammasome inhibitor, β-OHB protects the heart by enhancing autophagy and reducing inflammation and ROS production (non-oxidative effects). Elevated β-OHB by fasting, exercise, exogenous supplement, and SGLT2i treatment may enhance these protective effects and might be promising preventive measures or therapies for heart diseases β-OHB: β-hydroxybutyrate, SGLT2i: sodium-glucose cotransporter-2 inhibitors, Nlrp3: Nod-, LRR- and pyrin domain-containing protein 3, I/R: ischemia/reperfusion.

Despite the advances in the prevention, diagnosis, and treatment of cardiovascular diseases, they remain to be the leading cause of hospitalization and mortality. Close attention has been paid to the myocardial metabolism and increased utilization of β-OHB during cardiac stresses, such as heart failure and I/R injury. Besides its role as an energy source, β-OHB may have beneficial effects on the myocardium by inhibiting HDAC activity and Nlrp3 inflammasome formation and may play a vital role in the SGLT2i′s cardioprotection effects (Figure 3). Although most of the studies showed beneficial effects of mildly to moderately increased β-OHB in heart failure and I/R injury, there is some evidence that a prolonged exposure to or high blood levels of β-OHB are detrimental to the hearts. It is also noteworthy that the effects of β-OHB may depend on the formulation, dose, and delivery timing. Cardiac function is regulated by many signaling pathways affected by β-OHB, which makes it challenging to study. However, our efforts to further elucidate the mechanisms underlying the role of β-OHB during cardiac stresses may uncover a new era of heart failure and cardiac I/R injury management with more efficacious and safer therapeutics.
