# Supplementary material for: Beta-Hydroxybutyrate, Friend or Foe for Stressed Hearts
Source: Front Aging. 2021 Jun 8;2:681513. doi: 10.3389/fragi.2021.681513 (PMC8932950; doi:10.3389/fragi.2021.681513)
Supplement: Supplementary file 1 [file Table1.pdf]

Table 1. β-OHB studies in animals and humans

	Subjects	Therapies	Outcomes	Reference
**Animal studies**				
	Rats undergoing 20 min ischemia and 3 hr reperfusion	Fasting to increase β-OHB levels to 15.58 ± 2.56 mM/g	Fasting induced high levels of β-OHB reduced infarct size, total number of premature ventricular complexes and duration of ventricular tachycardia, and improved mitochondrial redox state	(Snorek et al., 2012)
	Rats undergoing 30 min ischemia and 2 hr reperfusion	Fed/fasted ± β-OHB with 25 µM/kg/min, IV	High concentrations of β-OHB reduced infarct size and apoptosis induced by I/R injury	(Zou et al., 2002)
**Beneficial**	Mice undergoing 30 min ischemia and 24 hr reperfusion	Delivery of β-OHB by 1.6 mM/kg/24 hr, SQ	β-OHB reduced infarct size, attenuated apoptosis and preserved cardiac function and improved mitochondrial function	(Yu et al., 2018)
	Dogs with progressive heart failure	Continuous intravascular β-OHB infusion, from 2.5 µM/kg/min to 5 µM/kg/min	β-OHB infusion preserved systolic function and ameliorated pathologic cardiac remodeling	(Horton et al., 2019)
	TAC/MI mice and post-MI remodeling rats	Ketone Ester diet increased ketone levels to 1.1 ± 0.2 mM/L in mice or 1.8 ± 0.2 mM/L in rats	Chronic oral supplement of Ketone Ester prevented and treated heart failure with attenuated	(Yurista et al., 2021)

			development of left ventricular dysfunction and remodeling	
	Six and 24-month-old rats	Fasting for 3 days or β-OHB 200 mg/kg/d IP	Starvation decreased ER stress and inflammasome formation, β-OHB treatment increased the expression of MnSOD and catalase	(Bae et al., 2016)
	Muscle-specific SCOT knockout mice	Fasting of SCOT knockout mice increased β-OHB	Elevation in β-OHB attenuated TAC-induced inflammation and macrophage infiltration	(Byrne et al., 2020)
	HFpEF mice by combining the age, long-term high-fat diet, and desoxycorticosterone pivalate challenge	1 mg/g/day Ketone ester diet or 10 μg/g/day Empagliflozin for 30 days	Increased β-OHB level attenuated Nlrp3 inflammasome formation and mitochondrial dysfunction and fibrosis	(Deng et al., 2021)
	Cardiomyocytes isolated from young (2.5 months), aged (2.5 years) and aged HF rabbits	2mM β-OHB treated for 24 hr	Elevated β-OHB levels can benefit mitochondrial repair in the aging heart	(Thai et al., 2019)
**Detrimental**	Isolated adult rat cardiomyocytes	5 mM β-OHB treat for 1, 4, or 16 hr	Prolonged exposure to β-OHB altered glucose uptake and increased ROS production	(Pelletier and Coderre, 2007)
	Rats	Ketogenic diet or β-OHB injection 100 mg/kg, Qod for 16 weeks	A ketogenic diet inhibits mitochondrial biogenesis and induces atrial fibrosis	(Xu et al., 2021)

Human studies				
**Beneficial**	Normal volunteers	1.6/3.2 mM/kg Ketone Esters drinks increased ketone levels to 2.8 ± 0.2 mM	β-OHB reduced blood glucose, free fatty acids, and triglyceride levels	(Stubbs et al., 2017)
	Normal volunteers	1.9 kcal/kg Ketone Esters increased ketone levels to 3.3 mM	β-OHB lowered plasma insulin, ghrelin, glucagon-like peptide-1, and peptide tyrosine levels	(Stubbs et al., 2018)
	Chronic HFrEF patients	β-OHB infusion 0.18 g/kg/h for 3 hr	β-OHB improved hemodynamics in HFrEF patients with increased CO, LVEF and MVO_2	(Nielsen et al., 2019)
	Type 2 diabetic patients with high cardiovascular risk	Empagliflozin or glimepiride for 30 days	Empagliflozin increased β-OHB level and attenuated Nlrp3 inflammasome activation	(Kim et al., 2020)

β-OHB: β-hydroxybutyrate, I/R: ischemia/reperfusion, ROS: reactive oxygen species, TAC/MI: transverse aortic constriction/myocardial infarction, IV: intravenous infusion, SQ: subcutaneous infusion, IP: intraperitoneal injection, ER: endoplasmic reticulum, MnSOD: manganese superoxide dismutase, CO: cardiac output, LVEF: left ventricular ejection fraction, MVO_2: myocardial oxygen consumption
